# Developing and testing a digital harm reduction app for GBMSM engaging in chemsex: a feasibility study grounded in users' lived experiences

**DOI:** 10.1186/s12954-025-01338-1

**Published:** 2025-11-21

**Authors:** Carol Strong, Patricia Anne Joson, Poyao Huang, Chuan-Chih Chen, Chia-Wen Li, Yuan-Chi Tseng, Tsan-Tse Chuang, Huei-Jiuan Wu, Stephane Wen-Wei Ku

**Affiliations:** 1 Department of Public Health, College of Medicine, National Cheng Kung University, Tainan, Taiwan; 2 Institute of Health Behavior and Community Sciences, National Taiwan University, Taipei, Taiwan; 3 In the Know Marketing Ltd., Kaohsiung, Taiwan; 4 Center for Infection Control and Department of Internal Medicine, National Cheng Kung University Hospital, College of Medicine, National Cheng Kung University, Tainan, Taiwan; 5 AIMS Fellows, National Tsing Hua University, Hsinchu, Taiwan; 6 The Kirby Institute, University of New South Wales, Sydney, Australia; 7 Division of Infectious Diseases, Department of Medicine, Taipei City Hospital Renai Branch, Taipei, Taiwan

## Abstract

**Background:**

Chemsex, involving substances like methamphetamine, GHB/GBL, and mephedrone among gay, bisexual, and other men who have sex with men (GBMSM), poses HIV and health concerns. Despite existing integrated care programs, uptake remains low, highlighting a missed opportunity for self-help harm-reduction digital strategies. This study employs a collaborative design approach, integrating insights from the community on the app prototype and assessed usability to guide future interventions.

**Methods:**

The app prototype was built on two design principles: recovery as a continuum with evolving goals like harm reduction and temporarily not using, and harm reduction dimensions on drug-, sex-, and HIV-related harms. Features were integrated into UPrEPU, a user-centered app supporting HIV PrEP adherence through flexible dosing and personalized reminders. Users could set goals, such as reducing chemsex frequency, supported by tailored feedback. The app includes an emergency alert system, local resources for clean needles and HIV testing, and myth-busting for the chemsex community. GBMSM with recent chemsex experience tested the app between January–December 2024. A one-month evaluation followed using the mHealth App Usability Questionnaire (1–7 scale, lower score indicates higher usability) and an interview to assess usability and impact. Interview transcripts were analyzed based on design themes to gather users' reflections and suggestions for enhancing harm reduction features.

**Results:**

Twenty GBMSM aged 23–46 in Taiwan reported a positive perception of usability (mean = 2.14, SD = 1.15). However, users indicated that HIV PrEP functions were utilized more frequently than drug-related functions. Dynamic goal setting should be more intuitive and tied to lived experiences, reflecting one's physical and mental state. Inadequate social support led individuals to adopt self-reliance strategies, such as tracking HIV PrEP uptake, setting hydration reminders, and prioritizing self-care. This extended to managing cravings, adhering to dosage limits, and achieving control over their health. Users emphasized the need for tools to go beyond drug-related features, focusing instead on helping them plan their time and set broader life goals, supporting a more holistic approach to well-being.

**Conclusions:**

A collaborative, user-centered approach to app design shows promise for digital solutions addressing chemsex-related harms, including HIV prevention, with functions closely tied to lived experiences.

**Supplementary Information:**

The online version contains supplementary material available at 10.1186/s12954-025-01338-1.

## Introduction

Chemsex refers to the use of drugs such as methamphetamine, gamma hydroxybutyrate (GHB) / gamma butyrolactone (GBL), and mephedrone among gay, bisexual, and other men who have sex with men (GBMSM) to enhance sexual experiences—a practice increasingly facilitated by mobile dating apps [1]. Individual studies across Asian countries report varying prevalence rates for chemsex or related substance use among MSM. A meta-analysis estimated the pooled prevalence of chemsex among MSM in Asia at 19%, with methamphetamine being the most commonly used substance, showing a pooled prevalence of 16% (95% CI 9–22%) [2]. Given the relatively high prevalence, chemsex-related harms, including depression, anxiety, suicidal ideation, and increased vulnerability to sexually transmitted infections, particularly HIV [3–5], constitute an important public health issue that may exacerbate health inequities among GBMSM.

Besides general harm reduction services for people who use drugs, such as needle and syringe exchange programs, there are various services available for individuals who engage in chemsex, including safer sex education, psychological counseling, or community-based interventions [6, 7]. In Taiwan, dedicated chemsex-focused services, such as support groups and mental health counseling, are available but remain underutilized, with only 10.5% of GBMSM chemsex users reporting service use [8], leaving a significant gap in meeting the needs of this population. Barriers to accessing services include insufficiently trained healthcare professionals, limited chemsex-specific services, and restrictive legal frameworks that criminalize drug use [9–11]. Stigma surrounding chemsex and drug use further obstructs service access. Criminalization creates a context where seeking help is stigmatized, deterring open discussions and reducing individuals' willingness to seek support [10]. Furthermore, many individuals perceive themselves as recreational users, reducing their motivation for behavioral change [12]. Nevertheless, even those who view their chemsex behavior as manageable may experience subtle negative impacts over time that they have not yet recognized. Therefore, chemsex should be understood as a dynamic and potentially escalating process, underscoring the necessity for accessible, early intervention strategies. Digital interventions may offer a particularly effective means to overcome some of these barriers by providing discrete, stigma-reducing, and geographically accessible support.

Digital interventions can be an effective means to support individuals early on, including those who may not perceive a need for help. These interventions are especially accessible to chemsex users who are already engaged with similar technologies to connect with others in the same behavior. Moreover, unlike traditional substance use clinics, where stigma may prevent individuals from seeking help, digital interventions offer a safe and anonymous space, reducing the discomfort and judgment often associated with seeking support [13]. Technology-based interventions have been extensively applied in the fields of ubstance use, and sexual health [14–16]. In the context of chemsex, mHealth programs have been developed to target harm reduction, such as supplying safe drug-use equipment [17], supporting MSM in minimizing chemsex-related harms and promoting reasoned participation [18], and providing educational interventions to reduce sexual harms [19]. However, these mHealth tools were developed based on an understanding of the specific harm reduction practices and needs unique to each chemsex context [17, 18], overlooking that harm reduction interventions must be tailored specifically to the target population.

In response to these identified needs, we developed a prototype harm reduction module that was integrated into an existing HIV pre-exposure prophylaxis (PrEP) adherence app, UPrEPU [20]. UPrEPU has been available in Taiwan since 2022, with hundreds of downloads and active users, and was selected because it was co-designed with GBMSM and supports complex PrEP dosing with reminders and logging features [20]. As part of the prototype, we also included an antiretroviral therapy (ART) diary with functions comparable to the PrEP log, allowing chemsex users living with HIV to track ART adherence and receive reminders.

The prototype module was designed to offer flexibility and adaptability, tailored specifically to users' personal goals, whether temporary cessation, abstinence, or harm reduction. By embedding these harm reduction strategies into the UPrEPU platform, the module is intended to promote consistent and effective HIV prevention behaviors among chemsex users. The prototype includes behavior regulation strategies, educational resources addressing drug-use risks, overdose prevention and management, and guidance on correct PrEP intake. Collectively, these preliminary features aim to mitigate potential harms and reduce the negative health and social impacts associated with problematic chemsex behaviors.

Using a user-centered design approach [21, 22], this study prioritizes users’ needs and lived experiences are prioritized throughout the design process. This study is situated in the prototyping phase of the harm reduction module and can inform the iterative design of the app, where the prototype is used to gather feedback from users to refine the design. Our aims were twofold: One, to describe the development process and core functions of the prototype harm reduction module, and two, to evaluate usability and collect user feedback via qualitative interviews to identify challenges and guide iterative refinement.

## Methods

### Design principle and theoretical underpinnings

The development of a digital solution for chemsex harm reduction is based on two major design principles. First, it adopts the concept of recovery as a continuum of outcomes, recognizing that individuals can take various paths toward addressing their drug use [23]. Recovery does not have to align solely with the traditional goal of abstinence. Instead, it may include a range of changes, such as temporarily stopping drug use or continuing while adopting practices to reduce the risks of harm or overdose. These goals are not fixed and can evolve over time; for instance, an individual may initially aim for abstinence but later shift toward harm reduction strategies as their circumstances and priorities change [24].

Second, it acknowledges chemsex harm reduction as a process that addresses multiple forms of harm, including drug-related, sex-related, and HIV-related risks, at different stages—before, during, and after chemsex [1]. Drug-related harm reduction focuses on managing intoxication and preventing overdose, emphasizing the importance of close monitoring and timely emergency intervention to avoid life-threatening situations. While HIV-related and sex-related harm reduction involves strategies such as PrEP to prevent the transmission of HIV and other sexually transmitted infections (STIs), as well as post-exposure prophylaxis (PEP) to address potential exposure following sexual activity.

By integrating these two theoretical foundations, the prototype is designed to address the diverse needs of individuals engaging in chemsex, supporting them in navigating their unique recovery journeys while reducing the associated risks.

### Prototype development

**Fig. 1:**
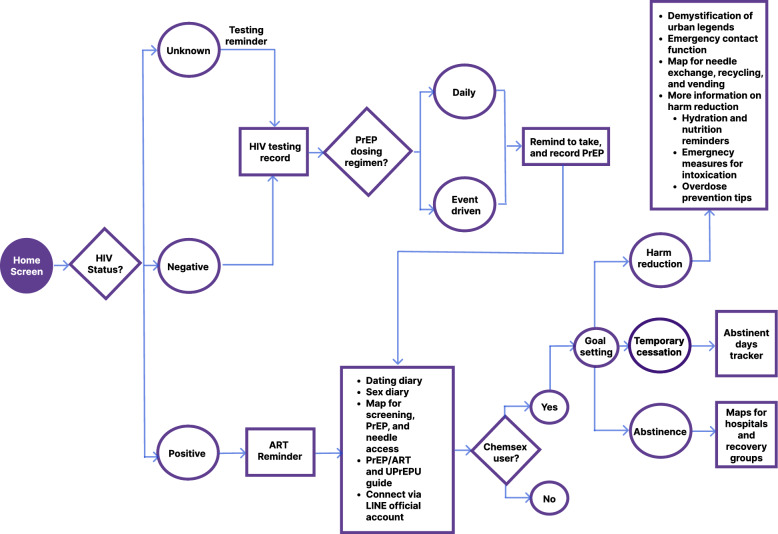
UPrEPU user flow. *HIV* Human Immunodeficiency Virus, *ART* Antiretroviral Therapy, *PrEP* Pre-exposure Prophylaxis

The design process involved an iterative approach, incorporating harm reduction principles into the app's existing framework. Development began on February 8, 2023 (scoping and technical planning); an iOS beta were delivered on August 21, 2023; an Android beta on October 6, 2023; and the harm-reduction upgrade was released on January 8, 2024. The multidisciplinary team comprised public health and human–computer interaction researchers who co-led design, content, and evaluation; the UPrEPU technical lead; and an external digital marketing and user-experience consultancy that supported community outreach. Pre-launch formative input was obtained via semi-structured interviews from key populations, GBMSM who engage in chemsex, to collect feedback, enabling the refinement of features and ensuring that the app prioritized functions deemed most essential by its users. Figure 1 presents the user flow of the prototype.

#### Goal setting

Harm reduction principles were incorporated to cater to the diverse goals of chemsex users, acknowledging that not all users aim to quit. The app offers three goal options: abstinence, temporary cessation, and harm reduction. Each goal is accompanied by specific guidance on what it entails. For abstinence, users are encouraged to make significant life changes, build new social networks, and develop coping strategies for managing negative emotions without relying on recreational drugs. For temporary cessation, the app allows users to track the number of days they have refrained from engaging in chemsex. For harm reduction, the focus is on balancing safety and pleasure, helping users minimize risks while continuing their behavior. Users can switch between goals at any time, ensuring flexibility and personalization to their needs (Supplementary file 1).

#### Harm reduction features

To prevent potential influence on individuals who do not engage in chemsex, harm reduction tools were embedded as an optional feature in UPrEPU, accessible only after user activation. The harm reduction module comprises essential elements aimed at promoting safer practices and reducing risks associated with chemsex, organized into HIV-related, sex-related, and drug-related components (Table 1).

**Table 1:** Features and harm reduction elements of the UPrEPU application

Types	Features	UPrEPU	Harm reduction features
Drug-related	Nutrition and Hydration		*During chemsex*: Provide reminders to stay hydrated and supplement nutrition after taking drugs
Drug-related	Intoxication and overdose management		*During chemsex*: (1) Send alerts to emergency contact (2) Providing information on what emergency measures can be taken in case of intoxication
Drug-related	Rest and self-care		*After chemsex*: (1) connect to hospitals and recovery group if user wants to stop engaging in chemsex (2) for users who want to temporarily stop, provide a calendar to track the number of days abstinent
Drug, HIV, Sex-related	Needle and syringe programmes		*Before chemsex*: Provide a map to look up places where there is needle exchange, recycling, and vending machines
Drug, HIV, Sex-related	Information, education and communication	(1) how to take PrEP/ART and use UPrEPU (2) Provide LINE official account and email for communication (3) Map to locate screening centers, PrEP access, and needle vending machines	*Before chemsex*: (1) Provide information on chemsex urban legends and demystify it (2) provide information on how to avoid overdose
Drug, Sex-related	Conscious planning	Dating diary, which allows users to record meet up details and timing, and partner HIV status	
HIV, Sex-related	Voluntary counselling and testing	Record HIV testing	
HIV, Sex-related	PrEP	Prompt notifications and record taking of PrEP (PrEP diary)	
HIV, Sex-related	Condom and lubricant use	Record whether using condom in sex diary (can also record sexual activities and experiences in the sex diary)	
HIV, Sex-related	Suppressive ART	Remind user to take ART	

##### HIV- and sex- related harm reduction

Recognizing that many chemsex individuals are concerned about HIV, we expanded UPrEPU—a self-monitoring app initially designed to support PrEP adherence—to address the broader needs of chemsex users. In short, UPrEPU includes a sex diary, allowing users to document sexual experiences, such as timing, STI prevention methods, sexual roles, and satisfaction levels [20, 25]. This integrates with a PrEP diary that records dosing schedules and provides tailored reminders based on the user’s regimen—whether daily or event-driven. For daily PrEP users, reminders prompt them to take one antiretroviral pill every 24 h. For event-driven users, reminders guide them to take a double dose 2–24 h before sexual activity, followed by single doses 24 and 48 h after. Beyond PrEP adherence, the app also provides a tracking function for antiretroviral therapy (ART) for individuals living with HIV. Additional features include educational resources on PrEP and HIV and a GPS-enabled map for locating nearby HIV testing centers.

##### Drug-related harm reduction

The app also addresses drug-related risks through several features. First, the app provides educational resources that debunk common myths and promote safer drug use practices. These resources tackle issues such as the use of inaccurate measuring tools, like bottle caps, which can lead to overdosing, and clarify the dangers of polydrug use.

Second, the emergency contact alert system allows users to set up trusted contact, such as a friend, before engaging in chemsex activities. Users can specify a time for the app to send an SOS, and if they do not cancel the alert by the set time, the app will automatically send a message to the contact via text or email. This system ensures that a support network is notified in case of an emergency, adding an extra layer of safety.

Third, a GPS-enabled map helps users locate essential harm reduction services, including needle exchange programs, recycling centers for safe disposal of drug paraphernalia, and vending machines offering clean needles and other necessary supplies. This locator ensures that users have access to critical resources that can reduce the risks associated with chemsex.

Fourth, the app features a collection of practical harm reduction articles that provide valuable information on maintaining hydration and nutrition during prolonged drug use and sexual activity, as well as strategies for managing substance use to minimize harm. Overall, these harm reduction features aim to support users in making safer choices while engaging in chemsex, enhancing their autonomy while prioritizing their health and safety (Supplementary file 2).

#### Functions for abstinence and temporary cessation

For users aiming for abstinence, the app features a GPS-enabled map connecting them to nearby support groups and rehabilitation centers. Those seeking temporary cessation can use a calendar to track abstinent days.

### Study procedures

The harm reduction upgrade of the app was officially launched on January 8, 2024. GBMSM with recent chemsex experience tested the app between January-December 2024. Participants first completed a baseline survey, followed by a one-month follow-up survey to assess the app's usability and feasibility. Subsequently, qualitative interviews were conducted to align the lived experiences of chemsex users with the app's functionalities and to gather detailed feedback for identifying areas for improvement.

#### Recruitment

Participants were recruited from an integrated care clinic specializing in sexual health and chemsex, from current UPrEPU users, and through online channels and word of mouth.

#### Eligibility criteria

Individuals were considered to be eligible to participate in this study if they are: (1) above 18 years old, (2) cisgender men who have sex with men or transgender female who have sex with men, (3) have a history of drug use (ecstasy, ketamine, crystal methamphetamine, mephedrone, and/or GHB/GBL) in a sexualized context in the previous year, (4) can read and understand Chinese; and (5) willing to communicate with the research group via the mobile messenger app, Line.

#### Onboarding and baseline survey

Eligible participants were invited to download and register on the UPrEPU app. Upon onboarding, participants completed baseline surveys capturing sociodemographic information (age, education, employment status), chemsex-related substance use, sexual behaviors (including condomless anal intercourse, number of sexual partners, history of STIs), preventive health behaviors (HIV status, PrEP usage, mpox vaccination, HPV vaccination, doxy-PEP), and utilization of chemsex-related health services.

Self-efficacy for sexual safety was measured using seven items rated on a five-point Likert scale ranging from "strongly disagree" (1) to "strongly agree" (5). Example items include "I can choose safer sex with a man I have sex with regularly" and "I am confident that I can have safer sex even if my partner really doesn’t want to" (Cronbach’s alpha = 0.72). Total scores ranged from 7 to 35, with higher scores indicating greater self-efficacy for safe sex [26].

Sexual satisfaction was assessed using four questions rated on a seven-point scale ranging from "not at all" (1) to "extremely" (7). Participants responded to statements reflecting their sexual lives, such as “In most ways, my sexual life is close to my ideal” (Cronbach’s alpha = 0.97) [27].

#### Follow-up survey

App usability was evaluated at a one-month follow-up using the mHealth App Usability Questionnaire [28]. The original scale consists of 21 items across three dimensions: ease of use and satisfaction, arrangement of system information, and efficiency, rated on a seven-point Likert scale from 1 (extremely strongly agree) to 7 (extremely strongly disagree), with an overall Cronbach’s alpha of 0.912 [28]. Given that our app was not designed for communication with healthcare staff, we removed four questions related to such interactions, resulting in a 17-item scale. Mean scores were calculated for each participant, with lower scores indicating better usability.

All survey responses are anonymized, and participants' answers are linked to an ID number created from a combination of parts of their Taiwan ID and birthdate.

#### Qualitative interviews

After the follow-up survey, a subset of respondents were invited to participate in an online qualitative semi-structured interviewto explore lived experiences with chemsex. During the interviews, the harm reduction upgrade of UPrEPU was introduced as a tool to facilitate discussions about participants’ chemsex journey and to gather suggestions for app improvements. The interview topics revolved around individuals’ chemsex recovery journey, their experiences with using the UPrEPU app, and their suggestions for its improvement. Each interview lasted between 45 and 60 min and was audio-recorded with prior consent from participants. Transcription were generated with the Plaud Note AI voice recorder, and transcripts were later reviewed by the research team for accuracy and to ensure context-specific cues are preserved.

Interview transcripts were imported into Excel for thematic analysis. All transcripts were translated into English, with both the original Chinese and English versions displayed side by side during coding to ensure accuracy and preserve linguistic nuance throughout analysis. Deductive coding was conducted by a single coder, focusing on aspects of participants’ chemsex journey, harms experienced, perception of app features in relation to their chemsex practices, and recommendations for app improvements. Additionally, to ensure rigor and minimize bias, the coder regularly consulted with the rest of the research team to review coding decisions and interpretations.

#### Participant compensation

Participants received e-vouchers as reimbursement for their time: 200 New Taiwan Dollars (NTD) for completing the baseline survey, 300NTD for the follow-up survey, and 500NTD for participating in qualitative interviews.

##### Data management

All survey and interview responses were anonymized, and participants' answers were linked to an ID number created from a combination of parts of their Taiwan ID and birthdate.

## Results

Twenty-five eligible participants were enrolled and completed the baseline survey, of whom twenty completed the follow-up. Among these twenty participants (Table 2), 35% were under 35 years old, most had a bachelor's degree or higher (80%), and the majority were employed (90%). The majority reported using methamphetamine (95%) for chemsex, with other commonly reported substances including Viagra (70%), GHB/GBL (55%), and alkyl nitrites (55%). High-risk sexual behaviors were prevalent; nearly all participants (95%) reported condomless anal intercourse in the past three months, and 30% had six or more male partners during that period. Preventive behaviors varied, with over half living with HIV (55%), 30% using PrEP, and high uptake of mpox (70%) and human papillomavirus (HPV) (65%) vaccinations. Participants reported moderate levels of self-efficacy for sexual safety (mean = 21.30, standard deviation = 7.73) and sexual satisfaction (mean = 4.48, standard deviation = 1.81). Half of participants had recently utilized chemsex-related health services such as therapy groups or mental health care. At the one-month follow-up, the app received positive usability feedback (ean = 2.14, standard deviation = 1.15).

**Table 2:** Participant characteristics, chemsex-related behaviors, preventive practices, and app usability evaluation

	Total N = 20
	N (%)
Baseline	
Sociodemographic characteristics	
Age < 35 (vs. ≥ 35)	7 (35.0)
Education: bachelor’s degree or higher	16 (80.0)
Employed	18 (90.0)
Type of substance used for chemsex	
Methamphetamine	19 (95.0)
GHB and GBL	11 (55.0)
MDMA	4 (20.0)
Viagra	14 (70.0)
Alkyl nitrites (Rush, poppers)	11 (55.0)
Sedative	4 (20.0)
Sexual behaviors	
Having a sexual partner living with HIV in past 3 months	9 (45.0)
Numbers of male sexual partners in the past 3 months ≥ 6	6 (30.0)
CLAI in past 3 months	19 (95.0)
Self-reported STI in the lifetime (any kind)^1^	7 (35.0)
Preventive behaviors	
HIV status and PrEP use	
HIV negative not on PrEP	3 (15.0)
HIV negative on PrEP	6 (30.0)
Living with HIV	11 (55.0)
Mpox vaccination uptake	14 (70.0)
HPV vaccination uptake	13 (65.0)
Doxy-PEP use experience	2 (10.0)
Chemsex services utilization in the past three months	
Chemsex recovery therapy groups or counseling	9 (45.0)
Mental health clinic/hospital or rehab center	9 (45.0)
Chemsex care programs affiliated with NGO	4 (20.0)
Needle Syringe Program	2 (10.0)
Self-efficacy for sexual safety^2^	21.30 (7.73)
Sexual satisfaction^2^	4.48 (1.81)
One-month follow-up	
mHealth app usability^2^	2.14 (1.15)

*GHB* gamma-hydroxybuturate, *GBL* gamma-butyrolactone, *MDMA* 3,4-methylenedioxymethamphetamine, *CLAI* condomless anal intercourse, *STI* sexually transmitted infection, *HIV* human immunodeficiency virus, *PrEP* pre-exposure prophylaxis, *HPV* human papillomavirus, *Doxy-PEP* postexposure prophylaxis with doxycycline, *NGO* nongovernmental organization

^1^STI includes gonorrhea, syphilis, genital warts, genital herpes, amoebic colitis, chlamydia, shigellosis, and hepatitis virus infection (including type A, B, and C virus)

^2^Data are presented as mean and standard deviation

Thirteen participants completed qualitative interviews. The qualitative findings highlight that participants’ lived experiences of social disconnection, self-regulation, and dynamic emotional state shifts directly influence how they engage with the UPrEPU prototype. Accordingly, every theme first presents participants’ lived experiences as critical context, and then shows how these experiences shaped perceptions of the app’s usability and functionality. Together, these insights point toward design refinements such as enhanced self-regulation support, and adaptive goal setting tools that are responsive to user’s fluctuating states.

### Social disconnection and the and the limits of emergency support

Social networks serve as both a gateway into and a pathway out of chemsex. On one hand, chemsex is often introduced through environmental influences, such as invitations from acquaintance, the influence of partners, or the impact of triggering events, which reinforce participants' engagement with the practice. On the other hand, severing ties with social networks can serve as a key exit strategy for those seeking to abstain from the practice, highlighting the importance of distancing oneself from environments that promote or normalize the behavior.

I had strong intentions every time. I deleted all the apps, cut off all my friends who used, blocked my dealers—completely erased everything. I did it very thoroughly. – Participant 13, age 41

However, this severing of ties is a double-edged sword. As participants distance themselves from their previous environment, many are left isolated and without social support. This context influenced why the app’s emergency contact function was perceived as unusable. Several participants expressed that they would not use the emergency feature, as disclosure to non-user friends risked stigma or misunderstanding.

Because using is a very private matter for me. I don’t want others to know. Meth is still considered an illegal drug. If I set a non-user friend as my emergency contact, they’d probably just get annoyed. They wouldn’t understand that meth is addictive and that it’s not a one-time thing—it’s something that keeps happening. For them, it’s like, "Why are you doing this again?" Eventually, they’d either give up on me, get mad, or just accept it but with negative emotions. So, I wouldn’t want to put them in that position. I wouldn’t want normal friends to know I use. – Participant 13, age 41

Because the circle is so small, and everyone is afraid of being betrayed. Because in fact, you will always see what is going on with the people around you. – Participant 05, age 34

At the same time, participants found little emotional support within chemsex networks. Relationships within these circles were described as transient, superficial, and lacking meaningful support. The influence of drugs particularly was perceived to diminish users’ capacity for empathy and care, making mutual support within these networks unreliable. As a result, trust—whether in friends or fellow chemsex participants—became a significant concern.

Yes, they [other chemsex users] only focus on their own feelings at that moment [during chemsex] and won't care about others. – Participant 06, age 31

Yes, or you become his buddy, or maybe, maybe, you may have something useful, and then you are someone you trust, but that is very rare because everyone really just doesn’t talk about it, I think. It's like a passer-by. Most of the time I just pass by, and then I never contact you again. – Participant 04, age 23

Overall, the statements of participants highlight a usability gap, as the current emergency contact feature depended on social trust that participants did not feel they had. As a result, participants usage of the prototype was often limited to its HIV-related functions, rather than its broader harm reduction or emergency features.

These findings underscore a critical gap in support for individuals attempting to disengage from chemsex. While severing ties with chemsex-related social circles is often a necessary step, the resulting isolation highlights the urgent need for non-judgmental, accessible support systems. Without alternative social networks, individuals seeking to exit chemsex are left vulnerable, emphasizing the importance of stigma-free resources that foster meaningful social and emotional connections.

### Self-regulation and the role of digital support in chemsex management

In the absence of adequate support systems, several individuals engage in self-regulation strategies to manage their chemsex practices. When asked which prototype features they valued, participants did not highlight available functions, such as myth debunking articles, chemsex hydration and nutrition guidance, or GPS-enabled maps for needle exchange and support services. Instead, respondents returned to describing their own self-regulatory practices—such as setting limits on one’s drug intake, controlling spending on drugs, and engaging in aftercare—underscoring that digital tools grounded in self-monitoring and personal accountability resonate more strongly and have higher usability than static information or service locators. As such, participants’ reflections illustrate how personal strategies remain central to their harm reduction efforts.

Every time. I almost always use it [meth] between 10-20cc, and I won’t go beyond that. I heard and heard from people that it would be scary if it was serious, so I didn’t dare. -- Participant 02, age 32

What I do is keep a record every time I use it, how much money I spend, and how many days I used it. I think seeing the usage helps. If I used it for a few days before, it might not feel as intense. But when I start spending money, that's when it hits, because I started keeping track of my expenses. In the past few years, I've been keeping track and realized that, looking back over a year, I might have spent over a hundred thousand on it. -- Participant 01, age 39

Yeah. When I use [meth], I always make sure I know how to take care of my body. – Participant 13, age 41

In many ways, individuals acted acted as their own form of personal support system in the absence of external care. This inclination toward self-reliance was also reflected in feature requests for the prototype. Rather than generalized information, participants proposed that the app should actively assist with basic self-care tasks, such as staying hydrated and resting after chemsex—functions that extend or reinforce their existing self-regulation strategies.

Sis, I forgot to tell you—how to save oneself during chemsex, such as in cases of methamphetamine overdose, severe dehydration from forgetting to drink water, or encountering irrational and dangerous online acquaintances who engage in stalking, threats, or intimidation. – Participant 03, age 40

Some participants even viewed the app as a lifeline, suggesting it should integrate direct connections to emergency services like 119 [the equivalent of 911 in Taiwan], given that chemsex participants often struggle to rely on one another for help. In this way, the participants envisioned the app to not only support self-regulation but to also function as a neutral and non-judgmental "friend"—one that could be trusted when others could not.

Beyond harm reduction, digital technology emerged as a substitute for traditional social support, offering a structured and consistent means of reinforcement. By integrating features that help users manage their chemsex engagement, the app can both facilitate self-regulation and reinforce autonomy, by enabling individuals to maintain a sense of control. At the same time, by acting as a trusted “friend” in moments of vulnerability, the prototype will be able to address gaps left by limited or unreliable social networks.

### Dynamic and holistic approach to digital interventions for chemsex management

While self-regulation and digital tools play a crucial role in managing chemsex engagement, interventions must also account for the dynamic and fluctuating nature of an individual's life. Currently, the app is structured around goal setting, enabling users to transition between abstinence and harm reduction. However, some participants perceived formal goal setting as a source of psychological pressure, as unmet goals could create a sense of failure rather than progress.

Emotional status and external influences can shift rapidly—often within seconds—making rigid goal setting approaches insufficient. Even when allowing for flexibility, setting goals may fail to align with users’ real-time experiences, potentially leading to frustration, self-blame, or disengagement. As one participant expounded on why they did not agree with the goal setting feature:

Hmm, let me think. There's a perspective that says whether it's pausing or harm reduction, it ultimately still moves towards the goal of continued use. So, if we define it this way, it almost feels like a declaration, which carries a certain heaviness and pressure. It's like making a pledge, but that pledge can lead to feelings of failure later if it doesn’t work out. -- Participant 09, age 39

This concern becomes especially relevant in the context of trigger events, which can rapidly alter an individual’s state of mind and substance use decisions. At one moment, a person may feel committed to abstinence, but after experiencing a stressor such as anxiety, their immediate need may shift to harm reduction. While the app allows for goal adjustments, it does not fully account for the unpredictability of emotional fluctuations, potentially reinforcing feelings of discouragement when users struggle to meet their stated goals.

To address this, participants emphasized the need for a more adaptive and responsive system that provides support based on immediate emotional needs rather than fixed milestones:

So, if I’m about to use it, and I open the app, the information inside should cater to different user states. For example, if I’ve been abstinent for two months and am now under pressure, like I was last week, the most important or helpful thing for me in that moment is the information related to harm reduction. Because that’s the kind of support or information I need most at that point.

However, let’s say a month or two later, I might be in a honeymoon phase or at the end of it. In that case, if I look at the harm reduction content, it might trigger certain associations that could lead me to relapse. So, at that point, what I need to see immediately when I open the app is something like how many days, I’ve been abstinent. Something that focuses on that kind of progress instead of triggering harm reduction-related thoughts. – Participant 09, age 39

Given the variability in emotional states, participants also emphasized the importance of tracking stressors and their role in shaping substance use patterns. Many realized that chemsex engagement was not simply a reaction to isolated events but rather the result of an accumulation of multiple stressors over time. Several participants expressed interest in features that could track trigger events, allowing for early intervention before a crisis occurs.

Hmm, that’s something you’d have to consider. But for me personally, I think it would help if I could mark significant events, maybe using colors, or tagging emotions like happiness or sadness. That would be useful for me. Before I joined Matrix [an outpatient substance addiction treatment program [29]], I thought using substances was just… like, if I was happy or if my boyfriend was upset because his boss scolded him, then I’d use. But after joining Matrix, I realized that substance use is actually the result of many small things accumulating.

Like, when I relapsed in May 2021, I knew it was because, in the months before that, my grandmother passed away. What I mean is, if I can keep track of these important events, I can recognize the buildup leading to a relapse. It’s like a point system—if I reach 10 points, I relapse. So, for example, my grandmother’s passing might be. -- Participant 12, age 38

The location is just to let me know that I might want to stay away from that place in the future because being close to it might remind me of past usage experiences. -- Participant 10, age 33

Incorporating features such as event tracking, emotional state monitoring, and location-based alerts could enhance the app’s ability to support self-awareness and relapse prevention. By helping individuals identify cumulative stressors, such features could offer real-time feedback and tailored coping strategies before a crisis occurs.

In addition to relapse prevention, participants emphasized that for interventions to be sustainable, they must extend beyond harm reduction and abstinence-focused strategies. Sustainable recovery is not just about reducing substance use but also about fostering healthier daily habits, social stability, and long-term personal goals.

Many participants described how engaging in structured activities and maintaining meaningful routines helped reduce their chemsex frequency.

Yeah, to be honest, I don’t really know what the app helps me with, but I hope it can serve as a reminder. It can remind me when to stop using the drug, or how many days I should stop using it, or it can remind me that I should take medicine, or do something else. Or if I want to record something today, I would think of it as a notebook or a diary. – Participant 10, age 33

Features, huh? When I first tried to stop using, and even now, I think that if I can successfully distract myself, it helps a lot. Like, when I really have cravings, if I can just shift my attention—because usually, when I get cravings, I’m just sitting on my bed, scrolling on my phone. There’s always someone to talk to on Grindr, people asking if I want to meet up, and sometimes I’ve already made plans. But if I can remove myself from that situation—if I can just get out of bed, put on my running shoes, and go outside for a run—then I feel like I can get through the day. – Participant 12, age 38

The frequency is quite high when I have nothing to do. If I am very focused on work, I actually won’t think about using these things. –Participant 05, age 34

Thus, for digital interventions to be effective and sustainable in chemsex management, it must move beyond goal setting and instead adopt a flexible, real-time support system that aligns with users’ immediate emotional needs. Incorporating event tracking, stress monitoring and relapse prevention tools can enhance self-awareness and provide timely, personalized interventions. Moreover, addressing broader life aspects—such as structured routines, self-care, and social stability—ensures that recovery is not solely about abstinence but about fostering long-term well-being and resilience.

## Discussion

This study explored how lived experiences can inform the design of digital interventions for chemsex harm reduction. We developed and tested a mobile app prototype aimed at supporting users in managing chemsex practices, including harm reduction and PrEP adherence. Our findings highlight that lived experiences play a crucial role in intervention design, and that the gap between user needs and app design needs to be addressed. Participants emphasized that their needs are dynamic and situational, shifting with emotional states and external factors. This finding underscores the importance of tailoring interventions to real-time needs, and adopting flexible, user-centered design approaches that adapt to evolving user experiences. Building on these findings, the next step is to refine the app’s features based on user feedback from this feasibility study, prioritize functions aligned with different stages of chemsex journey, and co-create additional tools with stakeholders to enhance usability, engagement, and reach. This iterative development process will help ensure that the app provides tailored harm reduction support and remains responsive to evolving community needs.

Lived experiences can directly inform intervention design by mapping out users experiences comprehensively. Our findings highlight how social isolation shapes users’ engagement with digital interventions and reinforces the need for app functions that respond to immediate needs, such as emotional state monitoring and location-based alerts. While users acknowledged that goal setting could be dynamic, they emphasized that the design should be more responsive to their current state rather than fixed goals, reflecting a gap between the original design and users needs. This reinforces the importance of user-centered and co-design methodologies, which inherently prioritize understanding and incorporating lived experiences. For instance, Yuan, Tseng, and Strong [30] argue that technological interventions for PrEP should respond to the multi-level realities of users’ lived experiences, including individual, interpersonal, and sociocultural dimensions, and propose human-centered design strategies accordingly. Moreover, Claborn et al. developed a digital platform aimed at improving community responses to overdose and prevention [31], highlight the importance of integrating community perspectives into harm reduction digital platforms. Similarly, the Budd app, specifically developed for chemsex harm reduction, actively involved potential users throughout all stages of the intervention planning process [18, 32]. These non-judgmental approaches and inclusive approaches are particularly important in the context of harm reduction for drug use [33].

There is growing recognition of the multifaceted nature of harms associated with chemsex, prompting the need for broader interventions. Similar to our prototype that encompassed drug, HIV and sex related harm reduction measures, the Budd app addresses multiple dimensions, including drug harm reduction, safer event planning, access to support services, adherence to HIV medication or PrEP, and providing support during chemsex events [32]. Gautam (2023) suggests mobile apps for chemsex harm reduction could incorporate features addressing drug-related harms, HIV/STI risks, and mental health support [17]. We find that a more holistic approach to chemsex management includes reminders for daily life activities beyond drug use, such as staying hydrated and maintaining regular routines, which is broader than the original design in the prototype. Additionally, Herrijgers (2022) highlights chemsex’s broader impacts, such as relational, financial, and occupational factors, indicating awareness of harms beyond health risks alone [18].

Our app in this paper, UPrEPU, incorporates harm reduction features specifically aimed at promoting safer practices and reducing risks associated with chemsex, structured into HIV-related, sex-related, and drug-related components. However, evaluating these multifaceted interventions can be challenging due to varied outcomes, necessitating the development of concise measurement tools that account for interconnected harms.

Although our study participants were not entirely disconnected from chemsex harm reduction services—nearly half had participated in chemsex recovery groups or counseling and exhibited high preventive behaviors such as vaccine uptake and PrEP usage—they still demonstrated greater social isolation than anticipated. This was evident from difficulties participants reported in identifying someone to assist them in emergencies. Such social isolation persists despite participants attending chemsex recovery groups in person. "Chosen family" members, defined as family-like networks of friends and current or former romantic partners, play a crucial role as sources of social support within the gay community [34], highlighting the importance of developing and applying these networks in harm reduction interventions. Therefore, digital solutions should include components designed to help individuals identify their social networks and activate functional social support.

This study has some limitations. The small sample size may limit the generalizability of the findings; however, the results provide a valuable foundation for further development. Most participants had prior experience with chemsex-related in-person interventions. While this experience allowed them to reflect on and compare in-person and digital solutions, it may limit the generalizability of the findings to individuals without such experience. Our digital solution was designed primarily to engage individuals with prior experience seeking chemsex help programs or practicing harm reduction. Whether this digital solution also appeals to those who avoid services entirely warrants further investigation. Additionally, the short follow-up period may have limited the ability to capture long-term needs and patterns, as some issues related to chemsex management may only emerge over months or years. Social desirability bias may have also influenced the positive feedback reported in the quantitative assessment of usability.

Our study demonstrates that digital solutions hold significant potential for chemsex harm reduction. A collaborative, user-centered approach to app design shows promises for digital solutions addressing chemsex-related harms, including HIV prevention, with functions closely tied to lived experiences and holistic well-being. This study underscores the potential of mHealth solutions to provide harm reduction support tailored to chemsex recovery stages, particularly for underserved populations.

## Supplementary Information

Additional file1 (DOCX 428 KB)

Additional file2 (DOCX 825 KB)

## Data Availability

The data generated and/or analyzed during the current study are not publicly available due to confidentiality concerns. However, data may be available from the corresponding author upon reasonable request, with the appropriate consent and under conditions ensuring participant confidentiality.
