# Supplementary material for: Developing and testing a digital harm reduction app for GBMSM engaging in chemsex: a feasibility study grounded in users' lived experiences
Source: Harm Reduct J. 2025 Nov 21;22:189. doi: 10.1186/s12954-025-01338-1 (PMC12639721; doi:10.1186/s12954-025-01338-1)
Supplement: Supplementary file 1 — Additional file1 (DOCX 428 KB) [file 12954_2025_1338_MOESM1_ESM.docx]

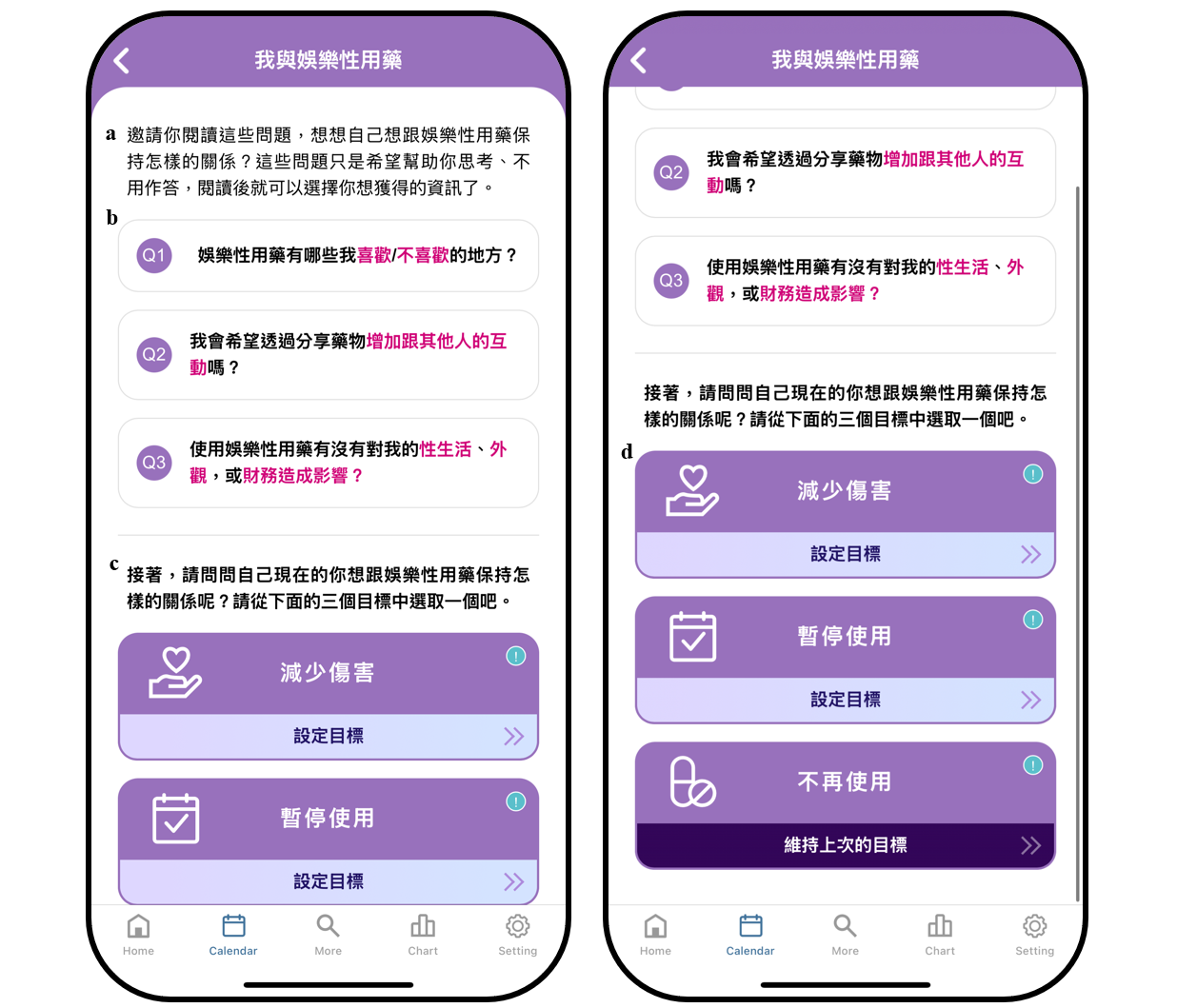


**Supplementary File 1: Goal setting feature of UPrEPU**

1. These questions are not meant to judge you but to help you think about your recreational drug use and whether it aligns with your goals. There are no right or wrong answers, and you don’t have to answer if you don’t feel comfortable.
2. Q1: Are there aspects of recreational drug use that you dislike or find unpleasant?

Q2: Do you hope to share your experiences to increase interaction with others?

Q3: Has recreational drug use affected your sex life, relationships, or financial situation?

1. Next, please consider how you currently want to manage your recreational drug use. Select one of the three goal options below.
2. (from top to bottom) harm reduction, temporary cessation, abstinence
