# Supplementary material for: Developing and testing a digital harm reduction app for GBMSM engaging in chemsex: a feasibility study grounded in users' lived experiences
Source: Harm Reduct J. 2025 Nov 21;22:189. doi: 10.1186/s12954-025-01338-1 (PMC12639721; doi:10.1186/s12954-025-01338-1)
Supplement: Supplementary file 2 — Additional file2 (DOCX 825 KB) [file 12954_2025_1338_MOESM2_ESM.docx]

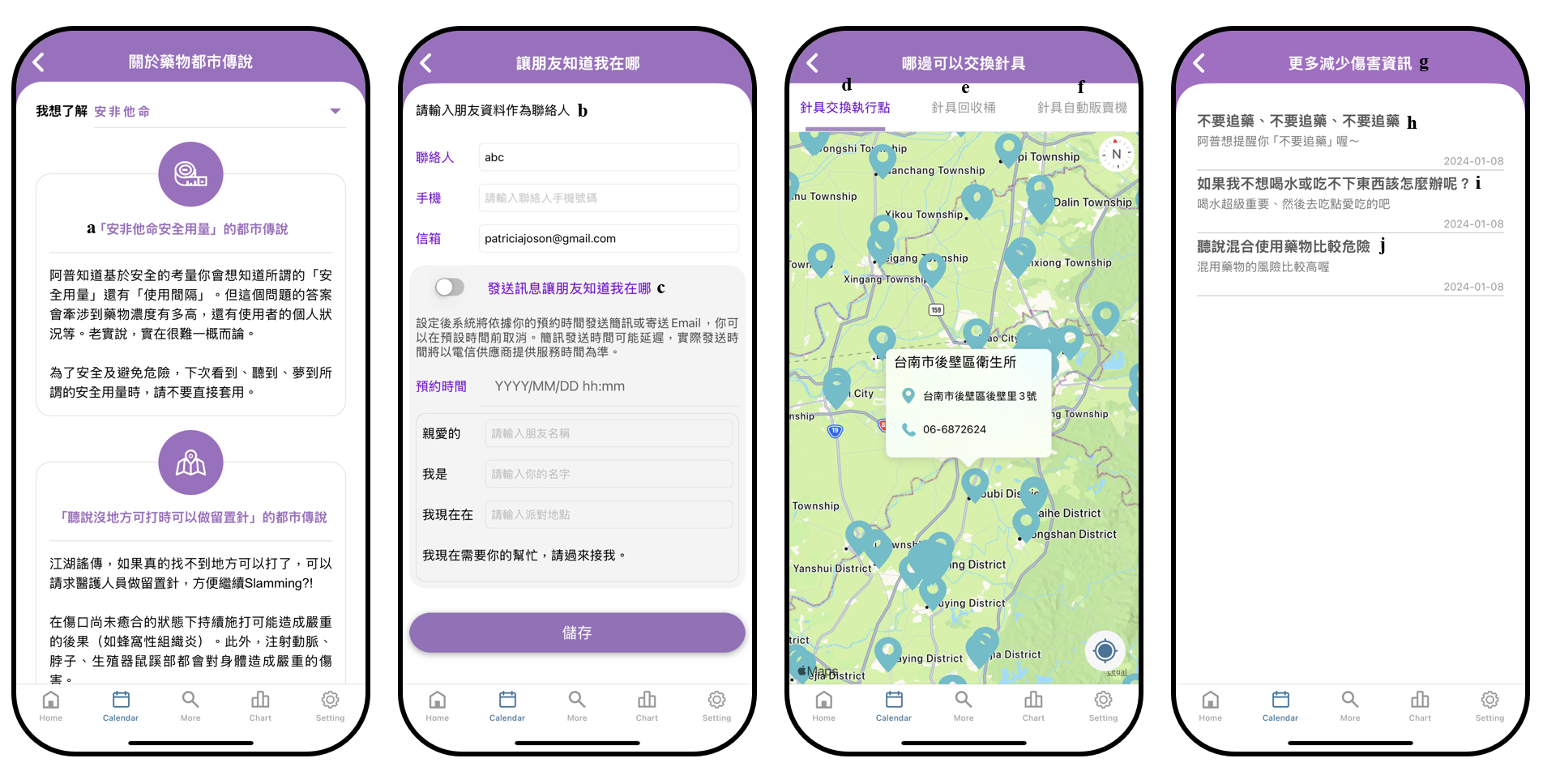


**Supplementary file 2: Four harm reduction functions of UPrEPU: (from left to right) (1) demystification of urban legends; (2) emergency contact function; (3) needle exchange map; (4) articles on harm reduction**

1. **The urban legend of "safe dosage" for amphetamines**

UPrEPU knows that for safety reasons, you might want to understand what is considered a "safe dosage" and the "interval between uses." However, the answer to this question depends on factors such as the concentration of the drug and the individual's condition. To be honest, it is difficult to give a one-size-fits-all answer.

For safety and to avoid risks, whenever you come across, hear about, or even dream of a so-called safe dosage, please do not apply it directly.

1. **Please enter your friend's contact information:**

Contact Person:
Mobile Number:
Email Address:

1. **Send a message to let your friend know your location**

Once set, the system will send an SMS or email based on your scheduled time. You can cancel before the scheduled time if needed. Please note that SMS delivery may be delayed, and the actual sending time will depend on the service provided by the telecom provider.

1. **Needle exchange sites**
2. **Needle recycling bins**
3. **Needle vending machines**
4. **More harm reduction information**
5. **Don’t chase the high, don’t chase the high, don’t chase the high!**

UPrEPU want to remind you “do not chase the high!”

1. **What if I feel thirsty or can’t eat?**
   Staying hydrated is super important, so drink plenty of water. If you’re having trouble eating, try something you enjoy!
2. **I’ve heard that mixing drugs is quite dangerous.**

The risks of mixing drugs are relatively high!
